# Case Report: Severe neonatal lupus in an infant with homozygous *NCF1* p.Arg90His variant and a *der*(14)*t*(4;14) translocation

**DOI:** 10.3389/fimmu.2026.1815781

**Published:** 2026-05-08

**Authors:** Xiaojing Yu, Jiwu Lou, Yuli Zhong, Lan Zhang

**Affiliations:** 1 Department of Pediatrics, Zhongshan City People’s Hospital, Zhongshan, China; 2 Prenatal Diagnosis Center, Dongguan Maternal and Child Health Care Hospital, Dongguan, China; 3 Department of Neonatology, Dongguan Maternal and Child Health Care Hospital, Dongguan, China

**Keywords:** der(14)t(4;14) translocation, genetic lesion, homozygous variant, NCF1, neonatal lupus erythematosus

## Abstract

Neonatal lupus erythematosus (NLE) is a rare, passively acquired autoimmune disease. The manifestations of NLE are varied, and the outcomes of the majority of cases result in spontaneous resolution. However, severe cases are limited. Emerging evidence highlights the genetic heterogeneity of NLE. The missense variant p.Arg90His in *NCF1* has been demonstrated to be associated with autoimmune diseases, including systemic lupus erythematosus. Chromosomal abnormalities manifest as developmental and growth delays and facial anomalies, among other phenotypic characteristics. Whether the co-occurrence of the missense variant p.Arg90His in *NCF1* and *der*(14)*t*(4;14) translocation would raise critical questions about the individual remains a mystery. In this report, we first describe a female patient with NLE who is homozygous for the p.Arg90His mutation in the *NCF1* gene and also exhibits a *der*(14)*t*(4;14) translocation. Whole exome sequencing (WES) identified a homozygous *NCF1* missense variant of NM_000265.4:c.269G>A(p.Arg90His) in the proband. Sanger sequencing confined the variant and showed that both parents were heterozygous for this variant. G-banding karyotype showed a chromosome abnormality of 47,XX,+*der*(14)*t*(4;14)(p15.2;q11.1) in the proband and a balanced translocation of 46,XX,*t*(4;14)(p15.2;q11.1) in her mother. The patient exhibited a refractory hematologic disease as a characteristic feature and was treated with glucocorticoids. She displayed unique facial features, developmental delay, and growth retardation during follow-up. This case offers a practical example to elucidate the genotype–phenotype correlations between chromosomal abnormalities, the *NCF1* variant, and the observed clinical manifestations. In addition, we propose that the homozygous *NCF1* p.Arg90His variant acts as a potent genetic modifier, exacerbating the severity of the clinical manifestations of NLE, with a predominant impact on the hematologic system.

## Introduction

Neonatal lupus erythematosus (NLE) is an autoimmune disorder acquired passively through the transplacental transfer of maternal autoantibodies. The predominant autoantibodies involved are anti-Sjögren’s syndrome-related antigen A (anti-SSA/Ro), anti-Sjögren’s syndrome-related antigen B (anti-SSB/La), antinuclear antibodies (ANA), and anti-U1 ribonucleoprotein (anti-U1-RNP) antibodies. Clinically, NLE manifests as a multisystem disorder with presentations ranging from congenital heart block and cutaneous lesions to hematological abnormalities, exhibiting diverse clinical expressions. Non-cardiac manifestations are generally more prevalent and reversible, attributable to the reduction in maternal antibody titers in the neonate within a few months postpartum. Conversely, cardiac involvement typically necessitates the implantation of a permanent pacemaker in affected neonates. Although maternal autoantibodies are the primary determinants, genetic variants significantly contribute to phenotypic variability (1). This variability is observed among non-identical twins exposed to similar levels of maternal antibodies (2, 3), implicating genetic differences as a modifier of the clinical phenotype.

Parental balanced translocations carry the risk of resulting in gametes with unbalanced karyotypes. The present patient had extra *der*(14) chromosomes consisting of proximal 4p and proximal 14q, inherited from a balanced translocation carried by the mother. The clinical manifestations of trisomy 4p vary, depending on the size and the location of the duplicated 4p segment and the accompanying monosomy of the partner chromosome (4). The characteristic phenotypes of trisomy 14 are developmental and growth retardation, minor facial anomalies, and multiple congenital anomalies (5). Our patient presents with unique facial features, developmental delay, and growth retardation. Her long-term outcome calls for our follow-up.

Here, we describe a Chinese female patient with severe NLE who harbors both a homozygous p.Arg90His mutation in the *NCF1* gene and a *der*(14)*t*(4;14) translocation, which is the first report in the English peer review literature. We compared the clinical and genetic profiles of the proband and the family members.

## Case presentation

**Figure 1:**
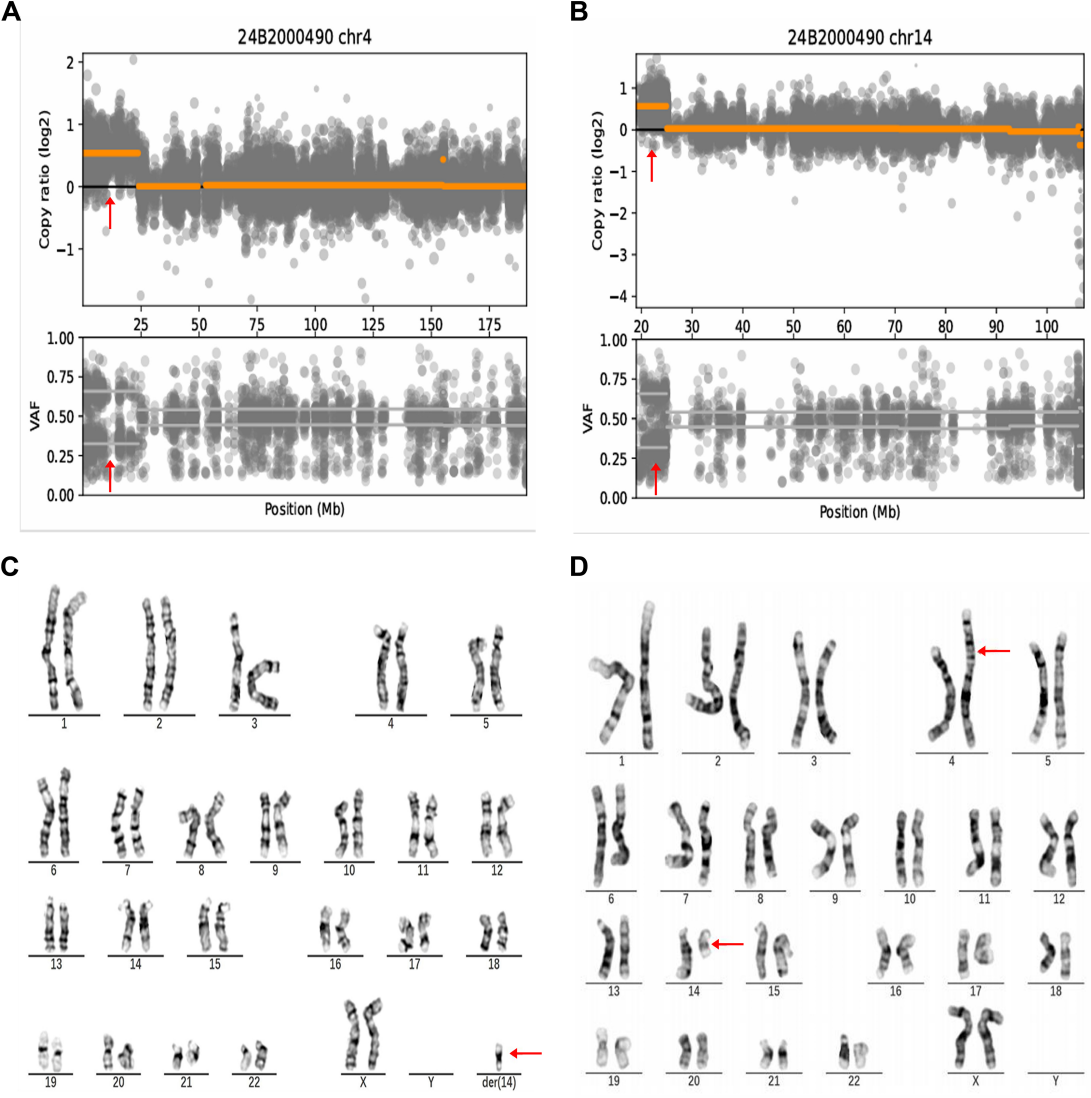
WES identified a 5.86 Mb duplication of 14q11.1q12(chr14:19000500-24977441, hg19) and a 23.88 Mb duplication of 4p16.3p15.2 (chr4: 10500-23891580, hg19) , the upper and lower subfigures respectively show the copy number ratio of two chromosomes (after log2 transformation, with a normal value of 0 and a copy number duplication region at ~0.58) and the allele depth ratio for heterozygous variants on the chromosomes (normal at~0.5, with duplication regions at 0.3 or 0.7). The duplication regions are indicated by red arrows. **(A, B)** .G-banding karyotype showed a chromosome abnormality of 47, XX, +der(14)(4;14)(p15.2:q11.1) in proband [**(C)** red arrowhead] and a balanced translocation of 46, XX, t(4;14)(p15.2,q11.1) in mother [**(D)**. red arrowheads].

**Figure 2:**
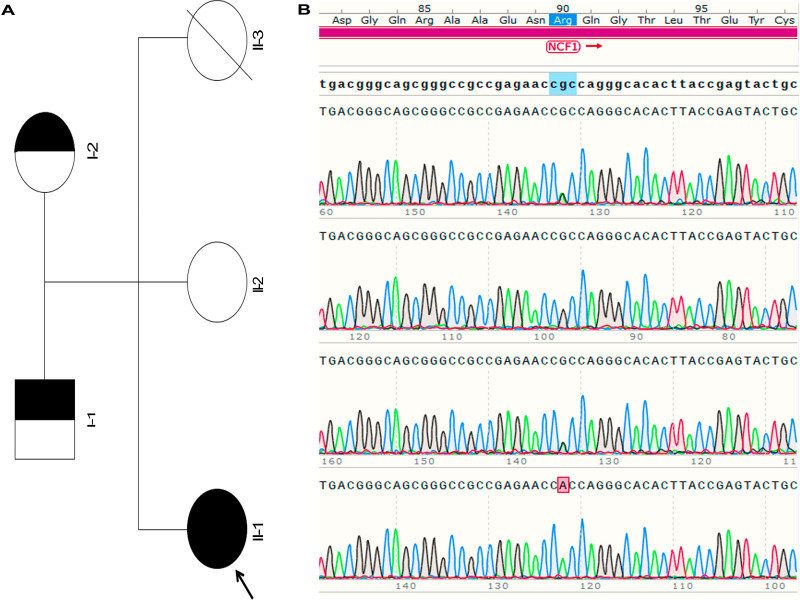
Pedigree and molecular analysis of the family. **(A)** The proband is indicated by an *arrow* in the pedigree. **(B)** The DNA analysis results for each family member are shown *on the right*. Sanger sequencing confirmed that the proband is homozygous for the *NCF1* variant c.269G>A (p.Arg90His), which was inherited from her carrier parents (father *I-1* and mother *I-2*). Her living sister (*II-2*) is wild type for this variant.

A 28-year-old woman, gravida 4, para 2, was admitted to the hospital due to threatened premature labor. She had a documented history of hypothyroidism, which was managed with oral levothyroxine treatment. Her firstborn daughter, delivered at full term, died several hours after birth due to coagulation disorders. Her second daughter was characterized as small-for-date infant. Diagnosed with cutaneous NLE at a different hospital, her skin lesions resolved spontaneously during the neonatal period. The mother delivered a female neonate via spontaneous vaginal delivery after experiencing a 2-week premature rupture of membranes at 33 weeks and 1 day of gestation. Weighing 1,370 g at birth, she was assessed to be a small-for-gestational age (SGA) infant. Her Apgar scores at 1, 5, and 10 minutes were 9, 10, and 10, respectively. She was admitted to the Neonatal Intensive Care Unit (NICU) due to very low birth weight. Physical examination revealed distinct facial features, including hypertelorism, a depressed nasal bridge, and microphthalmia. The initial blood routine revealed leukocytopenia (3.89 × 10^9^/L), mild anemia (120 g/L), and thrombocytopenia (10 × 10^9^/L). Empirical antibiotic therapy with penicillin (100 mg/kg per dose, IV, Q8h) and cefotaxime (50 mg/kg per dose, IV, Q8h) was initiated postpartum due to suspected neonatal infection. She experienced hypoglycemia, which was rapidly corrected. In subsequent hours, she developed blood stasis and ecchymoses. Cranial ultrasonography revealed no signs of intracranial hemorrhage. Echocardiogram detected patent foramen ovale (3.2 mm). The electrocardiogram (ECG) results were normal. Tests for bacterial culture, cytomegalovirus IgM antibody, herpesvirus DNA, and Epstein–Barr virus antibody all returned negative results. Metagenomic next-generation sequencing of blood samples identified *Acinetobacter baumannii* and *Klebsiella pneumoniae*, neither of which was deemed pathogenic in this context. The initial bone marrow aspiration revealed no abnormalities. Based on her family history and clinical presentation, NLE was suspected. Platelet infusion and intravenous immunoglobulin (IVIG) were administered. The mother was referred to a rheumatologist, who diagnosed her with asymptomatic systemic lupus erythematosus (SLE). The serological examinations of both the baby and her mother were positive for autoantibodies to SSA/Ro and SSB/La. ANA were identified at a rate of 1:320 (normal range, <1:80). Following the recurrence of symptomatic anemia and thrombocytopenia with active bleeding, particularly involving the skin and oral mucosa, prednisone therapy was initiated. Since the patient’s symptomatic anemia and thrombocytopenia recurred, prednisone treatment (1 mg/kg per dose, orally, Q12h) was administered. However, the dynamics of the blood laboratory indices revealed that the platelet count remained unstable and persisted at a low level; therefore, the dose of the prednisone treatment was adjusted to 2 mg/kg per dose. Over the next 5 days, the platelet count exhibited an upward trend, and the clinical symptoms were controlled; therefore, prednisone treatment was gradually discontinued. The dynamics of the blood laboratory indices and treatments are presented in **Table 1**. To identify whether gene mutations occurred in the family, whole exome sequencing (WES) was performed in the proband, which identified a 5.86-Mb duplication of 14q11.1q12 and a 23.88-Mb duplication of 4p16.3p15.2. Further G-banding karyotype showed a chromosome abnormality of 47,XX,+*der*(14)*t*(4;14)(p15.2;q11.1) in the proband and a balanced translocation of 46,XX,*t*(4;14)(p15.2;q11.1) in the mother (**Figure 1**). In addition, WES identified a homozygous *NCF1* missense variant of NM_000265.4:c.269G>A(p.Arg90His) in the proband (II-1). Sanger sequencing confirmed the variant and demonstrated that both parents (I-1 and I-2) were heterozygous carriers, whereas the proband’s sister (II-2) did not possess the variant (**Figure 2**). At the 5-month follow-up, the proband tested negative for ANA, SSA, SSB, and standard blood parameters. However, she displayed delayed motor development and failure to thrive. Her recorded weight and height were 5.8 kg and 58 cm, respectively, positioning both below the third percentile. She remained unable to lift her head or roll over.

**Table 1:** Dynamics of the blood laboratory indices and treatments of the proband.

Date	WBC (×10^9^/L)	HB (g/L)	PLT (×10^9^/L)	NE (×10^9^/L)	Treatment
10.13	3.89	123	10	1.66	–
10.13	9.76	169	29	4.59	IVIG (2.5g)
10.15	2.03	78	5	0.25	Blood and platelet transfusion
10.16	1.97	111	6	0.59	Platelet transfusion
10.18	3.42	129	61	1.02	–
10.19	2.86	105	23	1.15	Prednisone (1 mg/kg)
10.23	3.09	104	84	1.16	Prednisone (1 mg/kg)
10.25	3.10	104	57	0.93	Prednisone (1 mg/kg)
10.26	3.78	96	34	0.82	Prednisone (1 mg/kg)
10.27	3.61	88	25	0.83	Prednisone (2 mg/kg)
10.28	3.42	86	105	1.35	Prednisone (2 mg/kg)
11.06	3.79	124	16	1.38	Platelet transfusion, prednisone (2 mg/kg)
11.15	3.05	99	33	0.95	Prednisone (2 mg/kg)
11.17	3.52	100	54	0.88	Prednisone (2 mg/kg)
11.19	4.88	105	55	1.27	Prednisone (1 mg/kg)
11.21	4.77	93	69	1.66	Prednisone (1 mg/kg)

*WBC*, white blood cell count; *HB*, hemoglobin; *PLT*, platelet count; *NE*, neutrophil count.

## Discussion

NLE is a rare congenital autoimmune syndrome acquired through the transplacental transfer of maternal autoantibodies during pregnancy. Globally, NLE occurs in an estimated 6 per 1,000,000 live births annually (6). The incidence of NLE among mothers with positive autoantibodies is approximately 2%, and the risk increases by up to 20% if a previous child had a history of NLE (6, 7). The female-to-male ratio is similar. Notably, approximately 25%–60% of mothers carrying these autoantibodies are asymptomatic and unaware of their autoimmune status during pregnancy. However, up to 50% of these women may develop symptoms of SLE or Sjögren’s syndrome within 3–5 years postpartum (8). The persistence of maternal autoantibodies underscores the importance of early detection in mothers for timely evaluation, monitoring, and intervention, as infection and renal failure are the leading causes of mortality among these women (1). In our case, it remains uncertain whether the asymptomatic mother will progress to a symptomatic autoimmune disorder, highlighting the need for continued follow-up.

Common manifestations of NLE include cutaneous lesions, hepatic dysfunction, hematological abnormalities, and irreversible cardiac arrhythmias. Complete atrioventricular heart block, occurring either *in utero* or postnatally, represents the most severe complication, necessitating pacemaker implantation. Hydroxychloroquine treatment is recommended for mothers with positive autoantibodies and a previous history of cardiac NLE in a sibling to reduce the risk of the fetus developing cardiac NLE (6, 7). Given that non-cardiac manifestations are generally mild and self-limiting, the primary management strategy for the majority of NLE cases involves observation (6). In our case, the proband (II-1), without cardiac involvement, presented with refractory hematological disease as a distinguishing characteristic. The administration of glucocorticoids proved effective for the proband. Notably, approximately 5% of infants with NLE will progress to develop SLE during late adolescence or early adulthood (7). Therefore, children born to mothers with positive autoantibodies, including our patient, should undergo assessment for potential systemic autoimmune diseases and remain under specialist monitoring. In addition, siblings of the proband may also be at risk and warrant long-term observation for the potential development of systemic autoimmune conditions.

The missense variant p.Arg90His in *NCF1* (rs201802880), which was identified in 2017, is more prevalent among East Asians and rare among Europeans and South Asians. This variant demonstrates cross-ancestry associations with SLE, showing odds ratios (ORs) of 3.47 in Asians and 2.61 in Europeans, as well as associations with Sjögren’s syndrome (OR = 2.45) and rheumatoid arthritis (OR = 1.65) (9, 10). In our case presentation, the proband (II-1), with a homozygous mutation in *NCF1*, was diagnosed with NLE manifesting as refractory hematologic disease. In contrast, her mother (I-2), a carrier of the *NCF1* variant, remained asymptomatic, while her living sister (II-2), who does not carry the *NCF1* p.Arg90His variant, was diagnosed with cutaneous neonatal lupus. Similarly, a case study of a Chinese family showed that, while carriers of the *NCF1* variant (parents and brothers) remained healthy, twin sisters with a homozygous *NCF1* mutation exhibited symptoms of SLE (11). These observations suggest that, although the passive transfer of maternal autoantibodies is the primary trigger for NLE, there is significant phenotypic variability, indicating the role of genetic modifiers. While the exact pathogenesis of SLE remains unclear, exposure to environmental factors such as infections and toxins, in conjunction with genetic predisposition, may lead to its development. The *NCF1* gene, situated on chromosome 7q11.23, encodes p47^phox^, an integral component of the NADPH oxidase complex responsible for the generation of reactive oxygen species (ROS). These ROS are essential for microbial killing and immune regulation. Research confirms that the p.Arg90His mutation significantly increases the risk of SLE by reducing ROS production (9, 12). Experimental studies in *NCF1* R90H mouse models have demonstrated that the mutation reduces the NOX2-derived ROS production and exacerbates lupus through two complementary mechanisms: defective macrophage efferocytosis, which leads to aberrant T follicular helper (Tfh2) cell expansion and autoantibody production, and impaired ROS signaling in plasmacytoid dendritic cells, which promotes endosomal alkalinization, enhanced TLR7/9 signaling, and excessive type I interferon responses (13–15).

Risk alleles, whether single or involving multiple genetic variations, are strongly associated with the severity of lupus manifestations, including anti-dsDNA production, renal disease, age at diagnosis, and hematologic disorders (16). In both the present family and another reported family (11), patients with a homozygous mutation in *NCF1* presented with symptoms of refractory hematologic disease, especially thrombocytopenia. A carrier of the *NCF1* variant remained asymptomatic or healthy. Hematologic complications, including macrophage activation syndrome, have been documented in a 5-year-old girl carrying the homozygous p.Arg90His variant in *NCF1* (10). Therefore, we hypothesize that a mutation in the *NCF1* allele may exacerbate the severity of the clinical manifestations, with a predominant impact on the hematologic system, particularly affecting platelets. Further mechanistic insights derive from murine studies. Studies in murine models have verified that NCF1-R90H knock-in mice exhibit reduced macrophage efferocytosis and enhanced Tfh2 responses and that this variant promotes autoantibody production and kidney damage in both mice and SLE patients (17). In parallel, a 2018 study discovered that patients with hereditary p47^phox^ deficiency exhibit reduced platelet activation due to impaired oxidant species production by platelets (18). With impaired macrophage efferocytosis, along with heightened autoantibody production and immune dysregulation, platelet production declines and splenic clearance increases, making it difficult for the platelet counts to increase. Further experimental studies are required to elucidate the precise mechanisms.

In addition to the p.Arg90His variant in *NCF1*, the proband also carries an unbalanced chromosomal abnormality of 47,XX,+*der*(14)*t*(4;14)(p15.2;q11.1), resulting from a maternal balanced translocation: 46,XX,*t*(4;14)(p15.2;q11.1). This manifests as two large copy number duplications in the 14q11.1q12 and 4p16.3p15.2 regions, measuring 5.86 and 23.88 Mb, respectively. The protein-coding genes within the 4p16.3p15.2 region exceed 100 in number and include a potentially pathogenic 4p16.3 duplication region (chr4:331,568–2,010,962; ClinGen Triplosensitivity score 2). The associated clinical manifestations primarily include macrocephaly, tall stature, developmental and speech delay, and minor anomalies (19, 20). The 14q11.1q12 region contains more than 49 coding genes. The DECIPHER database shows one patient carrying a *de novo* duplication of approximately 1.01 Mb in the chr14:21,244,696–22,250,879 region. The main clinical features of this patient include posteriorly rotated ears, strabismus, highly arched eyebrows, cryptorchidism, micropenis, obesity, short stature, hypotonia, brachycephaly, microcephaly, aggressive behavior, speech delay, and intellectual disability (patient ID: 258583). Therefore, according to relevant guidelines for copy number variation (CNV) classification, both duplications can be classified as pathogenic CNVs. Furthermore, a supernumerary marker chromosome, *der*(14)*t*(4;14)(p15.31;q12) from a balanced translocation carrier mother, of similar size and chromosomal location, has been reported in two cases (21). Patient 1 was a 2-month-old girl presenting with persistent diarrhea, low body weight, palpebral fissures, low-set ears, and slender fingers and toes, with a normal cranial MRI. Patient 2 was a 9-year-old girl exhibiting growth retardation, microcephaly, intellectual disability, motor developmental delay, behavioral abnormalities, asymmetric eyes (left microphthalmia), and toe anomalies. The clinical manifestations in the present case (II-1), including developmental delay, growth retardation, hypertelorism, collapsed nose bridge, and small eye fissures, are likely related to these copy number duplications and warrant further follow-up on subsequent growth and development. However, it is neither well supported by evidence nor convincing that the chromosomal abnormality in II-1 contributes to her hematological anomalies. Within the 4p16.3p15.2 duplication region, the *NSD2* and *MSX1* genes are clearly haploinsensitive genes, which are associated with Rauch–Steindl syndrome and tooth agenesis, respectively. Within the 14q11.1q12 duplication region is the haploinsensitive gene *CHD8*, which is associated with intellectual developmental disorder with autism and macrocephaly. However, there is no evidence that duplication of this gene is associated with human diseases. In fact, neither of the two duplication regions contains clearly defined triplosensitive genes; in particular, there is no literature reporting on genes associated with abnormal hematological phenotypes. Furthermore, none of the previously reported patients carrying duplications in the corresponding regions exhibited hematological abnormalities similar to those of our proband.

Nevertheless, the maternal balanced translocation increases the risk of chromosomal abnormalities in the offspring. Theoretically, meiotic segregation patterns involving structurally abnormal chromosomes may give rise to gametes with segmental duplications or deletions in the offspring. Given the substantial size and pericentromeric location of the involved regions on chromosomes 4p and 14q, such imbalances are likely to be incompatible with survival. In this context, the early death of the first child (II-3), who presented with coagulation dysfunction, raises the possibility of a concomitant homozygous p.Arg90His variant and a more severe chromosomal abnormality. Unfortunately, neither WES nor chromosomal analysis could be performed to confirm this hypothesis.

## Conclusion

In conclusion, we report the clinical and genetic findings of a family affected by growth/developmental delay and NLE. Through a comparison of the clinical phenotypes and the underlying chromosomal and monogenic variants across family members, we have delineated the possible genotype–phenotype relationships. This family serves as a practical example for understanding how chromosomal abnormalities and the *NCF1* variant collectively contribute to the patient’s clinical presentation. We propose that the homozygousp.Arg90His variant in *NCF1* exacerbates the severity of the clinical manifestations of NLE, with a predominant impact on the hematologic system. This calls for further experiment for validation.

## Data Availability

The raw data supporting the conclusions of this article will be made available by the authors, without undue reservation.
